# Parental Culinary Skills and Children’s Eating Behavior in Brazil: A Cross-Sectional Study

**DOI:** 10.3390/nu18010051

**Published:** 2025-12-23

**Authors:** Thaís Souza dos Santos, Camila Ospina Ayala, Marina Zanette Peuckert, Carla Adriano Martins, Ana Maria Pandolfo Feoli, Micaella Bassanesi Bulla, João Pedro Soares Taffarel, Caroline Abud Drumond Costa

**Affiliations:** 1 Graduate Program in Pediatrics and Child Health, Pontifícia Universidade Católica do Rio Grande do Sul (PUCRS), Ipiranga Avenue, 6681, Porto Alegre 90610-970, RS, Brazil; nutrithaisantos@gmail.com (T.S.d.S.); marinazanette@hotmail.com (M.Z.P.); anafeoli@pucrs.br (A.M.P.F.); micaella.bulla@edu.pucrs.br (M.B.B.); j.taffarel@edu.pucrs.br (J.P.S.T.); caroline.drumond@pucrs.br (C.A.D.C.); 2 Collective Feeding Program, Institute of Food and Nutrition, Federal University Rio de Janeiro (UFRJ)—UFRJ-Macaé Multidisciplinary Center (CM UFRJ-Macaé), Carlos Chagas Filho Avenue, 373, Macaé 21941-902, RJ, Brazil; carlaadrianomartins@gmail.com

**Keywords:** food preparation behaviors, parenting, cooking, culinary skills, children’s eating behaviour questionnaire

## Abstract

Background: Childhood obesity is a persistent global health challenge, often rooted in early-life dietary patterns shaped within the home environment. Objective: To investigate the association between parents’ culinary skills, children’s eating behavior, and the degree of child involvement in family culinary practices. Methods: A cross-sectional, analytical study. In the public and private schools in southern Brazil. A total of 205 families with children aged 3 to 13 years participated. Parents or caregivers answered a structured questionnaire on culinary skills and sociodemographic variables. Children’s eating behavior was assessed through the validated Brazilian version of the Children’s Eating Behaviour Questionnaire (CEBQ). Student’s *T* test was used to compare means, and Pearson’s chi-square or Fisher’s exact test to compare proportions. Multivariate linear regression was applied to control for potential confounders. Analyses were conducted using SPSS version 27.0 and R software. Results: Most parents (90.7%) reported cooking regularly, and 65.9% involved children in cooking activities. The predominant culinary profile (40%) was classified as “convenience cooking,” marked by frequent use of processed ingredients. Healthier parental cooking practices were positively associated with adaptive eating behaviors in children, reflected by lower food fussiness, satiety responsiveness, food responsiveness and food refusal scores and higher enjoyment of food scores domains of the CEBQ. Conclusions: Despite the high prevalence of routine cooking, convenience-oriented practices remain dominant, reflecting broader sociocultural patterns. Engagement in healthier cooking practices was positively associated with more favorable eating behaviors in children. These findings underscore the importance of promoting culinary education and parental involvement in cooking as strategies to support healthy childhood eating behaviors.

## 1. Introduction

Obesity in children and adolescents is a global public health concern. According to a 2025 analysis based on data from the Global Burden of Disease study, updated estimates of overweight and obesity among children and adolescents aged 5 to 14 years revealed that, by 2021, approximately 93.1 million individuals in this age group were living with obesity (95% uncertainty interval (UI): 89.6–96.6 million) [1]. When obesity occurs during transitional stages such as childhood and adolescence, it often persists into adulthood and is associated with a range of cardiometabolic and psychosocial comorbidities, as well as increased risk of premature mortality [2].

In this context, promoting healthy eating behaviors from the earliest years of life has become a central strategy in prevention actions. Among these strategies, teaching culinary skills has emerged as a promising approach. A systematic review demonstrated that culinary interventions aimed at children and adolescents under 18 years of age resulted in significant reductions in body mass index (BMI) z-scores [3]. Teaching children to cook can promote a healthier lifestyle, facilitate more appropriate food choices and contribute to a positive relationship with food. Interventions focused on developing practical culinary skills among children and adolescents have proven to be relevant not only in preventing obesity, but also in addressing nutritional deficiencies and eating disorders [4].

In this scenario, it is essential to consider the role of the family, especially parents, as influential agents in children’s eating behavior [5,6,7,8]. This influence becomes even more critical given the alarming growth in the consumption of ultra-processed foods in several countries [6], a trend that has been attributed, at least in part, to the reduction in home meal preparation [5]. Such changes reflect the transformations inherent in the nutritional transition, characterized by less healthy eating patterns and greater industrialization of food [9].

In recent decades, there has been growing interest in research exploring the relationship between cooking skills, parenting practices and children’s eating behavior [10]. A study carried out in the urban area of Minas Gerais, Brazil, identified greater consumption of ultra-processed foods among children whose parents adopted practices such as physical force (*p* < 0.018), food reward (*p* < 0.002) and food restriction (*p* < 0.011). However, the study had limitations regarding the sample size and the instruments used to evaluate these practices [11]. In another study, conducted with a sample of 511 participants from Germany and France, and which used the Child Eating Behavior Questionnaire (CEBQ), an association was observed between negative parenting styles and inappropriate eating behaviors in preschoolers. Inconsistent parenting, in particular, had a direct impact on both food seeking and food avoidance. However, this study also had limitations regarding the assessment techniques used [12].

Considering that parental feeding practices can significantly shape children’s eating behaviors, they should be carefully targeted to promote a healthy and non-obesogenic food environment. It is important to emphasize that these practices are deeply rooted in cultural aspects, which require a sensitive and contextualized approach in studies of this nature. Thus, this study aimed to investigate the association between parents’ cooking skills, children’s eating behavior, and the degree of child involvement in family culinary practices.

## 2. Materials and Methods

### 2.1. Study Design

This cross-sectional study followed the Strengthening the Reporting of Observational Studies in Epidemiology (STROBE) statement [13].

### 2.2. Participants

Children and adolescents aged 3 to 13 years and their parents/caregivers were included in this study. Pregnant adolescents were not included in the study. Participants were recruited by convenience sampling from three public and private schools in the capital of southern Brazil. Parents and caregivers were approached during school hours, at meetings or events previously scheduled by the schools, and invited to participate voluntarily. Within each participating school, recruitment was conducted in a random and independent manner, without predefined selection criteria beyond having a child enrolled in the institution and within the target age range.

### 2.3. Setting

Data were collected in person using mobile devices. Parents/caregivers were randomly approached and invited to complete the questionnaires as long as they lived with the child/adolescent and were aware of their eating routines and habits. Only one parent/caregiver was required to complete the survey. Data were collected between May and September 2023, depending on the school’s availability. The research team consisted of dietitians, nurses, and previously trained undergraduate nutrition students.

### 2.4. Data Measurement

#### 2.4.1. Sociodemographic Variables

A questionnaire was administered for identification data of the parent/caregiver and the child/adolescent, such as age (years), sex, kinship, and self-identified skin color/race. Data such as level of education and family income were categorized according to the Brazilian Economic Classification Criterion of the Brazilian Association of Research Agencies [14].

#### 2.4.2. Children’s Anthropometric Measurements

Body mass and height were measured using standardized procedures, with participants barefoot and wearing minimal clothing, employing a calibrated digital scale and a portable stadiometer (AVANUTRI). Weight and height data were entered into world health organization (WHO) Anthro (0–5 years) and AnthroPlus (5–19 years) software to calculate z-scores for BMI-for-age and height-for-age [15]. Nutritional status was classified according to WHO growth standards and subsequently reclassified into two categories: non-overweight and overweight [16,17].

#### 2.4.3. Culinary Skills

The initial version of the instrument was created by two researchers based on national and international guidelines, such as the “Guidelines for Designing Age-Appropriate Cooking Interventions for Children” [18], the Food Guide for the Brazilian Population [19], and supplemented with results from previous studies in the area [20,21]. The content was subsequently reviewed by experts in the field to ensure relevance and clarity. The instrument was designed to capture the family’s usual cooking practices, including the frequency of cooking at home and the cooking tasks typically performed. Further details on each of these issues are presented in the following sections.

##### Questions About Cooking Practices

To explore cooking habits, participants were asked the following questions: “Do you have the habit of cooking?”, “On how many of the past 7 days did you cook lunch or dinner at home?”, and “What foods do you prepare most often?”. Emphasis was placed on preparing lunch and dinner as these are main meals and generally require more preparation.

##### Types of Food Preparation

The types of food preparation included options such as beans, grilled meat, frozen pizza, homemade bread/cake, microwave popcorn, and potatoes in an air fryer. These activities were selected based on the technique and skill level required, considering the act of cooking an essential practice and skill for promoting autonomy towards adequate diet and healthy eating [18,22,23].

##### Child Involvement and Feeding Practices

Additional questions addressed child involvement in the kitchen and family’s usual eating practices, according to the “10 steps for an adequate and healthy diet” in the Food Guide for the Brazilian Population [19]. Evidence-based culinary skill guidelines for children, adapted to different age groups [18], were developed considering their typical motor skills and the activities expected for each stage of development. Based on these guidelines, multiple-choice options were offered with various activities corresponding to each age group. This approach allowed us to assess the type of food-related tasks children were performing in the kitchen and determine whether they were age appropriate.

##### Consumption of Ultra-Processed Foods

Participants were asked about the frequency of ultra-processed food consumption by their child over the past 7 days based on the NOVA food classification system [24], which groups foods according to the nature, extent, and purpose of the industrial processing they undergo.

##### Assessment of Culinary Skills

Culinary skills were assessed based on 3 concepts from the literature that address practices and consumption [20]: Healthy cooking: Culinary techniques such as grilling and steaming, using natural seasonings, trying new recipes, and confidence in one’s cooking skills. Usual cooking: Weekly planning of meals and use of different culinary techniques, with weekly grocery shopping and confidence in one’s ability to prepare meals. Convenience cooking: Limited time spent planning or preparing meals, preference for quick snacks or ready-to-eat meals, and frequent use of artificial seasonings and microwave.

#### 2.4.4. Children’s Eating Behavior

Children’s eating behavior was assessed using the CEBQ. It consists of 35 items to be completed by the child’s primary caregiver, distributed across 8 subscales addressing the Interest in Food and Food Refusal domains: emotional overeating (eating more in response to negative emotional states); emotional undereating (eating less in response to negative emotional states); desire to drink (preference for beverages, particularly sugar-sweetened drinks); food responsiveness (eating in response to external food cues, such as the sight or smell of food); enjoyment of food (general interest in food and pleasure experienced when eating); satiety responsiveness (ability to recognize and adjust eating according to internal feelings of fullness); slowness in eating (tendency to eat slowly during meals); food fussiness (high selectivity or reluctance to try new foods). Each item is rated on a 5-point Likert scale, as follows: (1) never, (2) rarely, (3) sometimes, (4) often, and (5) always. In this study, we used the CEBQ version adapted for use in Brazil (CEBQ-BR), allowing for a more comprehensive screening and understanding of the eating behavior of children included in the study by using a tool adequately representing the reality of the population while still serving its original purposes [25].

### 2.5. Data Analysis

Descriptive analyses were conducted using mean and standard deviation (SD) for quantitative variables and absolute and relative frequencies for categorical variables. Comparisons between mean values were performed using Student’s *t* test, while differences in proportions were assessed using Pearson’s chi-square test with adjusted residual analysis or Fisher’s exact test, when appropriate. To examine the association between parental cooking type (healthy, usual, and convenience) and children’s eating behaviors (as assessed by the CEBQ domains), multivariate linear regression models were fitted. Each CEBQ domain was included as a continuous outcome, and cooking type was modeled as a categorical independent variable, using usual cooking as the reference category. The model was adjusted for parental education level and family income following recommendations from the literature [5,20,26]. Regression coefficients (β) with their corresponding 95% confidence intervals (95% CI) and *p*-values were reported. The significance level was set at 5% (*p* < 0.05).

Statistical analyses were performed using IBM SPSS Statistics, version 27.0. [27] and R software (version 4.3.3, https://www.r-project.org/ (accessed on 11 October 2025)) to illustrate the percentage distribution of children participating in culinary activities with adult supervision.

### 2.6. Ethical Considerations

The study was approved by the Research Ethics Committee of Pontifícia Universidade Católica do Rio Grande do Sul (PUCRS) and Porto Alegre Municipal Health Department (SMSPA), Brazil (approval number 5950233). All parents/caregivers participating in the study provided written informed consent. Children aged >6 years and adolescents who agreed to participate in the study provided written informed assent. Children aged ≤6 years or those who were illiterate received explanations about the study as described in a linguistically adapted version of the informed assent form and agreed verbally to participate.

## 3. Results

### Characteristics of the Study Population

Of the 215 parents and guardians initially eligible for the study, 10 were excluded for not completing the questionnaire in full, resulting in a final sample of 205 participating families. Regarding the sociodemographic profile of parents and caregivers, most self-identified as White (67.0%), and the most common familial relationship to the child was the mother (77.6%). In terms of education, higher education levels (undergraduate and graduate degrees) predominated among caregivers (63.0%). and the majority self-reported having a monthly family income > R$6060 per month (53%).

Regarding the characteristics of the children included in the study, the mean age was 7.7 ± 2.4 years, the majority were in the age range of 5 to 9 years (71%), male (53%), and 72% self-declared as being of white skin color. According to the BMI classification based on the WHO growth standards, 55.6% of the children were classified as not overweight. The mean z-score for height-for-age was 0.21 ± 1.11, and the mean z-score for BMI-for-age was 1.02 ± 1.37 (Table 1).

Regarding culinary skills (healthy, usual, and convenience cooking), 83 families (40%) were classified into the convenience cooking group, representing the most frequent type of cooking. In contrast, 65 families (32%) were categorized in the usual cooking group, and 57 families (28%) in the healthy cooking group. However, among these families, 87% reported that they consider it important to include and involve children in the kitchen, even though they do not do so. Of all 205 participants, 90.7% reported having the habit of cooking. When asked about the most frequent type of food prepared over the past 7 days, 60% reported having cooked beans. Regarding child involvement in cooking activities, 135 participants (66%) reported that they usually take the children to the kitchen and involve them in preparing meals. When asked about the importance of involving children in the kitchen from an early age, 88% agreed that it is important. When asked about the reasons preventing the child from getting involved, responses included fear, lack of time, perception of child’s inability, and concern about the mess. Data regarding the families’ culinary skills profile are described in Table 2.

Table 3 presents the results regarding the CEBQ domains and the eating styles of children and adolescents in the sample. The Interest in Food domain presented a mean of 2.86 ± 0.66, indicating a moderate tendency toward food-approaching behaviors, especially in the food responsiveness, desire to drink and enjoyment food subdomains. On the other hand, the mean for the Food Refusal domain was 2.90 ± 0.54, suggesting a moderate presence of food avoidance behaviors, especially in the food fussiness, satiety responsiveness, and slowness in eating subdomain.

Table 4 presents the results of the association of the habit of cooking, child involvement in the kitchen, and type of cooking with the CEBQ scales. Food refusal was the only factor showing a statistically significant association between those who have the habit of cooking and those who do not (*p* = 0.015). Regarding the habit of involving the child in the kitchen, enjoyment food (*p* = 0.032), the mean Food refusal domain score (*p* = 0.033), satiety responsiveness (*p* = 0.049), and slowness eating (*p* = 0.025) showed a statistically significant association between those who involved the child in the kitchen and those who did not. When analyzing the association between those who believe child involvement in the kitchen is important and the CEBQ scales, satiety responsiveness was the only factor showing a statistically significant association (*p* = 0.048). Regarding the type of cooking, desire to drink (*p* = 0.035), enjoyment food (*p* = 0.031), mean Food Refusal domain score (*p* = 0.016), satiety responsiveness (*p* = 0.004), and food fussiness (*p* = 0.026) showed a statistically significant association when comparing healthy cooking vs. usual/convenience cooking. Regarding the type of cooking practice, significant differences were observed across the groups in specific domains of eating behavior. Mean scores for the Food Refusal domain (*p* = 0.027), satiety responsiveness (*p* = 0.013), and slowness in eating (*p* = 0.026) differed significantly among children from families adopting healthy, usual, or convenience cooking practices. These findings suggest that children in families with healthy cooking practices may exhibit greater sensitivity to satiety cues and a slower, more deliberate eating pace, indicating potentially healthier self-regulation of food intake compared with children in the other two groups.

Regarding the type of cooking practice and its association with demographic variables, the analysis showed that although no statistically significant association was found in the global, the adjusted residual analysis revealed significant differences between some categories. A higher proportion of parents with college or graduate degrees was observed among those adopting healthy cooking practices (75.0%) compared with those following usual (52.0%) or convenience cooking practices (64.0%). Conversely, participants with elementary education were more frequent in the usual (20.0%) and convenience (20.0%) groups (Table 5).

For family monthly income, families in the healthy cooking group more often reported incomes above R$6060.00 (65.0%), whereas those in the usual (48.0%) and convenience (48.0%) groups were less represented in this range. Lower income levels (≤R$1212.00) were more common among the usual (32.0%) and convenience (25.0%) groups. No significant differences were found between cooking practices and the child’s age group. However, adjusted residuals indicated differences by sex, with a higher proportion of girls in the healthy cooking group (56.0%) and boys in the convenience group (63.0%) (Table 5).

In multivariate analysis, controlling for variables on level of education and income based on the literature, significant associations were observed between types of cooking and several CEBQ scales. Compared with the usual cooking group, participants with healthy cooking practices showed lower satiety responsiveness scores (β = −0.36; 95% CI: −0.61 to −0.11; *p* = 0.005), indicating that children from these families had a reduced sensitivity to internal satiety cues. Similarly, healthy cooking was associated with lower food fussiness (β = −0.43; 95% CI: −0.79 to −0.08; *p* = 0.018) and food refusal scores (β = −0.23; 95% CI: −0.42 to −0.03; *p* = 0.025), suggesting greater food acceptance among children. Conversely, higher enjoyment of food scores were found among families with healthy cooking practices (β = 0.33; 95% CI: 0.02 to 0.64; *p* = 0.037), indicating greater pleasure in eating behaviors within this group. No significant differences were observed for the convenience cooking group when compared to the usual cooking reference (Table 6).

**Figure 1:**
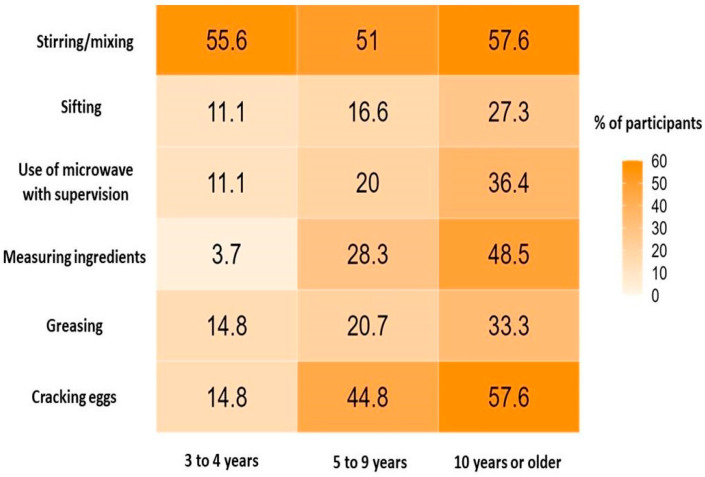
Culinary skills according to age group. Percentage distribution of children by age group in culinary activities performed with supervision. The tasks were categorized according to international recommendations for appropriate age culinary skills [18].

Child involvement in the kitchen was analyzed by age group, where those aged 9 years or older were more commonly involved in the kitchen (n = 53, 75.41%). Children aged 3 to 5 years were more adequately involved with regards to the type of tasks performed in the kitchen. Among the 17 children in this age group who used to assist in food-related tasks, 15 (88.23%) performed age-appropriate tasks (Figure 1).

## 4. Discussion

This study investigated the association between parents’ culinary skills, children’s eating behaviors, and their involvement in family cooking practices. Most parents were classified within the convenient cooking group. Healthier parental cooking practices were positively associated with adaptive eating behaviors in children, reflected by lower food fussiness, satiety responsiveness, food responsiveness and food refusal scores and higher enjoyment of food scores. Regarding child involvement in culinary activities, older children (≥9 years) were more frequently engaged in kitchen tasks, while younger children (3–5 years) participated less often but performed more age-appropriate activities.

According to the recommendations of the Brazilian Dietary Guidelines, healthy eating should be promoted through the encouragement of cooking “from scratch,” prioritizing the use of fresh or minimally processed foods, and moderate use of culinary ingredients [19]. In our study, however, most participants were classified within the convenience cooking group. This pattern may reflect broader sociocultural factors that favor the use of time-saving products, which help reduce both the physical and mental burden associated with planning and preparing meals [28,29]. Such behaviors are often aligned with the convenience and practicality offered by ultra-processed foods, despite their known negative impact on health [24].

Our findings showed that healthy cooking practices were associated with lower scores of food fussiness, food refusal, and satiety responsiveness, and with higher scores of enjoyments of food. This pattern underscores the importance of a supportive home food environment and active parental involvement in the development of adaptive eating behaviors in children [30,31]. These findings agree with the literature suggesting that cooking skills are associated with reduced consumption of ultra-processed foods and increased sensitivity to physiological hunger and satiety cues [21,32]. The literature also demonstrates that improved satiety responsiveness is negatively associated with childhood adiposity, reinforcing the notion that family food environments and parental modeling play a decisive role in shaping healthier eating behaviors [33].

In contrast, a study conducted with over 500 children in Alabama found that negative parental practices were associated with maladaptive eating behaviors, such as higher emotional eating, greater food responsiveness, more pronounced food demandingness, and higher satiety responsiveness and enjoyment of food, without any significant link between these behaviors and positive cooking practices. These discrepancies may reflect differences in sample characteristics and sociocultural contexts, suggesting that family eating dynamics are shaped by broader cultural and environmental factors [12].

Healthy cooking practices, mediated by increased confidence in cooking skills, were also associated with a lower likelihood of overeating and a greater degree of autonomy over food choices. This suggests that children exposed to a healthy and diverse range of foods tend to develop a health-conscious dietary repertoire and be less dependent on ultra-processed products, corroborating studies that highlight the importance of nutrition education from an early age to promote long-term health [21,32]. Similarly, a scoping review on hands-on meal preparation activities reported that these practices enhance positive perceptions of healthy foods, increase food acceptance, and foster healthier dietary behaviors among children, despite methodological variations across studies [34].

Although our results did not identify significant associations between sociodemographic variables and types of cooking practices, other studies point to the contrary. For example, a study conducted in Finland examined the association between parental feeding practices and children’s eating behaviors, suggesting that there is a significant relationship between these practices and the level of education and family income; socially disadvantaged neighborhoods showed a high tendency for unhealthy eating practices and higher body mass indexes in children, when compared to those living in more advantaged neighborhoods [35]. Another study suggested that families with a higher level of education and better socioeconomic status tend to make healthier food choices and present a higher frequency of preparing meals at home [20,36].

The nutritional transition observed in recent decades, characterized by increased consumption of ultra-processed foods and a reduced demand for natural foods and home meal preparation, reflects profound changes in family eating habits and lifestyles. These transformations have contributed to dietary patterns that favor the development of nutrition-related noncommunicable diseases [37]. According to the Nutrition Transition Model, the pace and nature of this process vary across countries and population subgroups. While most high-income countries, and an increasing number of low- and middle-income nations, are currently in advanced stages of this transition, the phenomenon can be attributed to multiple social and structural factors, including greater participation of women in the workforce, changes in traditional family structures, and time constraints that limit opportunities for cooking and the intergenerational transfer of culinary skills [38,39]. Such shifts have consequently reduced family influence on children’s eating behaviors. Nevertheless, as highlighted in the Nutrition Transition framework, this trajectory is not inevitable. With strong political commitment and the implementation of public policies that promote healthier food environments, it is possible to slow or even reverse the shift toward diets dominated by ultra-processed products [39].

The limitations of this study include its restricted representativeness, as the sample was drawn exclusively from the southern region of Brazil and recruited by convenience from a small number three schools located in the capital city. These factors may limit the generalizability of the findings to other regions or to the national level. Despite this limitation, we included children and adolescents from public and private schools covering different socioeconomic and ethnic groups for a broader representation of the Brazilian population. Furthermore, the topic addressed here involves different perspectives and is subject to a variety of beliefs, myths, and convictions that may have influenced the participants’ responses.

## 5. Conclusions

This study demonstrated that healthier parental cooking practices are positively associated with more adaptive eating behaviors in children, characterized by lower food fussiness, food refusal, and satiety responsiveness, and by higher enjoyment of food. In addition, older children tended to be more involved in cooking tasks, while younger children participated less frequently but engaged in activities appropriate to their developmental stage. These findings emphasize the importance of supporting families in developing healthier and more confident cooking habits as part of comprehensive strategies to foster positive eating behaviors in childhood. Encouraging parental engagement in home cooking, and the active involvement of children in meal preparation, may contribute to healthier food preferences, greater food autonomy, and the prevention of diet-related disorders across the lifespan.

## Figures and Tables

**Table 1:** Characteristics of the sample.

Variable	N = 205
**Parents and caregivers**	
Skin color/race—n (%)	
White	138 (67.0)
Black	43 (21.0)
Yellow (Asian descent)	2 (1.0)
Indigenous	1 (1.0)
Brown (mixed race)	21 (10.0)
Kinship—n (%)	
Father	33 (16.0)
Mother	159 (77.6)
Grandmother	6 (2.9)
Grandfather	3 (1.5)
Other	4 (2.0)
Level of education—n (%)	
No schooling completed	2 (1.0)
Elementary school	34 (17.0)
High school/Technical degree	39 (19.0)
College degree/Graduate degree	130 (63.0)
Monthly family income—n (%)	
Up to R$1.212	54 (26.0)
R$1.213 to R$3.636	24 (12.0)
R$3.637 to R$6.060	19 (9.0)
>R$6.060	108 (53.0)
**Children**	
Age (years)— mean ± SD	7.7 ± 2.4
Age group—n (%)	
3 to 4 years	27 (13.0)
5 to 9 years	145 (71.0)
10 years or older	33 (16.0)
Sex—n (%)	
Female	97 (47.0)
Male	108 (53.0)
Skin color—n (%)	
White	147 (72.0)
Black	33 (16.0)
Yellow (Asian descent)	2 (1.0)
Indigenous	0 (0.0)
Brown (mixed race)	23 (11.0)
BMI classification—n (%)	
Non-overweight	114 (56.0)
Overweight	91 (44.0)
Height-for-age (z-score)—mean ± SD	0.21 ± 1.11
BMI-for-age (z-score)—mean ± SD	1.02 ± 1.37

N = total number of participants; % = percentage; BMI = body mass index, SD = standard deviation.

**Table 2:** Culinary skills profile, as reported by parents.

Variable	N = 205 (%)
Has the habit of cooking	
Yes	186 (91.0)
No	19 (9.0)
Frequency of home cooking over the past 7 days	
1 to 2 days	40 (20.0)
3 to 4 days	47 (23.0)
5 or more days	118 (57.0)
Foods most often prepared	
Beans	123 (60.0)
Roast beef	64 (31.0)
Frozen pizza	2 (1.0)
Homemade bread/cake	10 (5.0)
Potato in an air fryer	6 (3.0)
Believes child involvement in the kitchen is important	
Yes	180 (88.0)
No	25 (12.0)
Has the habit of involving the child in the kitchen	
Yes	135 (66.0)
No	70 (34.0)
Tasks the child assist in the kitchen	
Stirring/mixing	108 (53.0)
Sifting	36 (18.0)
Greasing	45 (22.0)
Cracking eggs	88 (43.0)
Measuring ingredients	58 (28.0)
Using of the microwave (with supervision)	44 (22.0)
Common practices of the parent or caregiver	
Cook vegetables and greens	129 (63.0)
Encourages healthy choice	157 (77.0)
Involves the family in meal planning	84 (41.0)
Eats meals together around the dining table	145 (71.0)
Allows the child to use screens during meals	63 (31.0)
Encourages the child to participate in grocery shopping	112 (55.0)
Over the past 7 days, your child ate the following with your consent	
Chocolate and candies	88 (43.0)
Sandwich cookies	26 (13.0)
Packaged snacks	22 (11.0)
Pastries	16 (8.0)
Processed meat	28 (14.0)
Powdered juice/gelatin	18 9.0)
Ultra-processed foods	198 (97.0)
Type of cooking	
Healthy	57 (28.0)
Usual	65 (32.0)
Convenience	83 (40.0)

N = total number of participants; % = percentage.

**Table 3:** Children’s and adolescents’ eating styles as assessed by the CEBQ.

Variable	Range	N = 205
		Mean ± SD
Interest in food	1 a 5	2.86 ± 0.66
Emotional overeating	1 a 5	2.45 ± 0.87
Desire drink	1 a 5	2.67 ± 1.07
Food responsiveness	1 a 5	2.63 ± 0.93
Enjoyment of food	1 a 5	3.67 ± 0.86
Food refusal	1 a 5	2.90 ± 0.54
Satiety responsiveness	1 a 5	2.99 ± 0.68
Slowness in eating	1 a 5	3.02 ± 0.92
Food fussiness	1 a 5	2.97 ± 0.99
Emotional undereating	1 a 5	2.61 ± 0.89

CEBQ: Children’s Eating Behaviour Questionnaire; N = total number of participants; % = percentage; SD = standard deviation.

**Table 4:** Relationship between cooking habits and CEBQ score.

Variable	Has the Habit of Cooking		*p*	Has the Habit of Involving the Child in the Kitchen				*p*
	Yes	No		Yes		No		
	Mean ± SD	Mean ± SD		Mean ± SD		Mean ± SD		
Mean—Interest	2.87 ± 0.68	2.69 ± 0.46	0.127	2.89 ± 0.65		2.79 ± 0.68		0.295
Emotional overeating	2.46 ± 0.89	2.37 ± 0.70	0.655	2.48 ± 0.86		2.40 ± 0.89		0.549
Desire to drink	2.68 ± 1.08	2.61 ± 0.96	0.810	2.64 ± 1.06		2.73 ± 1.10		0.541
Food responsiveness	2.66 ± 0.95	2.31 ± 0.52	0.015 *	2.68 ± 0.92		2.53 ± 0.93		0.297
Enjoyment of food	3.69 ± 0.86	3.47 ± 0.85	0.291	3.77 ± 0.80		3.48 ± 0.94		0.032 *
Mean—Refusal	2.90 ± 0.53	2.93 ± 0.67	0.810	2.84 ± 0.48		3.02 ± 0.64		0.033 *
Satiety responsiveness	2.99 ± 0.66	3.06 ± 0.86	0.717	2.93 ± 0.66		3.13 ± 0.71		0.049 *
Slowness in eating	3.04 ± 0.92	2.83 ± 0.89	0.347	2.91 ± 0.90		3.22 ± 0.93		0.025 *
Food fussiness	2.95 ± 0.98	3.19 ± 1.13	0.310	2.88 ± 0.96		3.16 ± 1.04		0.054
Emotional undereating	2.61 ± 0.89	2.63 ± 0.94	0.935	2.63 ± 0.87		2.59 ± 0.94		0.783
**Variable**	**Believes child involvement in the kitchen is important**		***p***	**Type of cooking**				***p***
	**Yes**	**No**		**Healthy**	**Usual**		**Convivence**	
	**Mean ± SD**	**Mean ± SD**		**Mean ± SD**	**Mean ± SD**		**Mean ± SD**	
**Mean—Interest**	2.85 ± 0.67	2.91 ± 0.57	0.663	2.89 ± 0.62	2.89 ± 0.72		2.80 ± 0.64	0.630
Emotional overeating	2.45 ± 0.88	2.46 ± 0.82	0.989	2.55 ± 0.82	2.41± 0.88		2.42 ± 0.90	0.471
Desire to drink	2.62 ± 1.07	3.01 ± 1.01	0.088	2.44 ± 0.90	2.93± 1.17		2.62± 1.06	0.060
Food responsiveness	2.63 ± 0.98	2.63 ± 0.98	0.981	2.71 ± 0.96	2.62 ± 0.90		2.58 ± 0.93	0.651
Enjoyment of food	3.69 ± 0.84	3.54 ± 0.98	0.412	3.88 ± 0.78	3.60 ± 0.88		3.59 ± 0.89	0.177
**Mean—Refusal**	2.88 ± 0.52	3.01 ± 0.69	0.294	2.75 ± 0.48	2.95 ± 0.46		2.96 ± 0.62	0.027 *
Satiety responsiveness	2.96 ± 0.68	3.25 ± 0.69	0.048 *	2.77 ± 0.67	3.11 ± 0.62		3.06 ± 0.72	0.013 *
Slowness in eating	2.99 ± 0.89	3.25 ± 1.07	0.179	2.84 ± 0.95	2.92 ± 0.86		3.22 ± 0.91	0.026 *
Food fussiness	2.96 ± 0.98	3.05 ± 1.10	0.668	2.73 ± 0.91	3.08 ± 0.96		3.06 ± 1.05	0.076
Emotional undereating	2.63 ± 0.87	2.48 ± 1.05	0.423	2.68 ± 0.87	2.69 ± 0.86		2.51 ± 0.94	0.342

* *p* < 0.005; CEBQ: Children’s Eating Behaviour Questionnaire; SD = standard deviation.

**Table 5:** Demographic data and associations with the type of cooking.

Variable	Healthy	Usual	Convenience	*p*
Level of education—n (%)				0.056
No schooling completed	0 (0.0)	1 (1.5)	1 (1.2)	
Elementary school	4 (7.0)	13 (20.0) *	17 (20.0) *	
High school/Technical degree	10 (18.0) *	17 (26.0)	12 (14.0)	
College degree/Graduate degree	43 (75.0) *	34 (52.0) *	53 (64.0)	
Monthly family income—n (%)				0.295
Up to R$1212	12 (21.0)	21 (32.0)	21 (25.0)	
R$1213 to R$3636	3 (5.3)	8 (12.0)	13 (16.0)	
R$3637 to R$6060	5 (8.8)	5 (7.7)	9 (11.0)	
>R$6060	37 (65.0) *	31 (48.0)	40 (48.0)	
Age group—n (%)				0.660
3 to 4 years	6 (11.0)	10 (15.0)	11 (13.0)	
5 to 9 years	40 (70.0)	43 (66.0)	62 (75.0)	
10 years or older	11 (19.0)	12 (18.0)	10 (12.0)	
Sex—n (%)				0.057
Female	32 (56.0)	34 (52.0)	31 (37.0) *	
Male	25 (44.0)	31 (48.0)	52 (63.0) *	

* *p* < 0.005; n = number; % = percentage.

**Table 6:** Multivariate linear regression analysis of the relationship between type of cooking and CEBQ scales.

Outcome	Healthy Cooking		Convenience Cooking	
	b (95% CI)	*p* ^‡^	b (95% CI)	*p* ^‡^
Mean—Interest	0.13 (−0.09 to 0.34)	0.258	−0.05 (−0.25 to 0.15)	0.620
Emotional overeating	0.23 (−0.08 to 0.53)	0.144	0.04 (−0.24 to 0.32)	0.792
Desire to drink	−0.28 (−0.63 to 0.07)	0.116	−0.24 (−0.56 to 0.08)	0.135
Food responsiveness	−0.43 (−0.79 to −0.08)	0.018 *	0.01 (−0.27 to 0.30)	0.923
Enjoyment of food	0.33 (0.02 to 0.64)	0.037 *	−0.01 (−0.29 to 0.27)	0.955
Mean—Refusal	−0.23 (−0.42 to −0.03)	0.025 *	−0.01 (−0.19 to 0.17)	0.885
Satiety responsiveness	−0.36 (−0.61 to −0.11)	0.005 *	−0.05 (−0.28 to 0.17)	0.641
Slowness in eating	−0.07 (−0.40 to 0.26)	0.670	0.25 (−0.05 to 0.55)	0.106
Food fussiness	−0.43 (−0.79 to −0.08)	0.018 *	−0.04 (−0.36 to 0.29)	0.827
Emotional undereating	−0.04 (−0.36 to −0.28)	0.813	−0.21 (−0.50 to 0.08)	0.156

^‡^ adjusted for education level and income; b = regression coefficient (indicates the effect of cooking type on CEBQ domains); * *p* < 0.005; 95% CI = 95% confidence interval; CEBQ: Children’s Eating Behaviour Questionnaire; SD = standard deviation.

## Data Availability

The data presented in this study are available upon request from the corresponding author due to ethical restrictions.
